# Epigallocatechin-3-gallate inhibits osteogenic differentiation of vascular smooth muscle cells through the transcription factor JunB: Epigallocatechin-3-gallate inactivates the transcription factor JunB

**DOI:** 10.3724/abbs.2024060

**Published:** 2024-06-03

**Authors:** Tiantian Li, Fei Fang, Hongmei Yin, Zhen Zhang, Xiangxiu Wang, Erxiang Wang, Hongchi Yu, Yang Shen, Guixue Wang, Weihong He, Xiaoheng Liu

**Affiliations:** 1 Institute of Biomedical Engineering West China School of Basic Medical Sciences & Forensic Medicine Sichuan University Chengdu 610041 China; 2 West China School of Pharmacy Sichuan University Chengdu 610041 China; 3 Department of Cardiology the Third People’s Hospital of Chengdu Affiliated Hospital of Southwest Jiaotong University Chengdu 610000 China; 4 Key Laboratory for Biorheological Science and Technology of Ministry of Education State and Local Joint Engineering Laboratory for Vascular Implants Bioengineering College of Chongqing University Chongqing 400030 China; 5 JinFeng Laboratory Chongqing 401329 China; 6 Department of Physiology West China School of Basic Medical Sciences & Forensic Medicine Sichuan University Chengdu 610041 China

**Keywords:** medial arterial calcification, chronic kidney disease, epigallocatechin-3-gallate, vascular smooth muscle cell, osteogenic differentiation

## Abstract

Medial arterial calcification (MAC) accompanying chronic kidney disease (CKD) leads to increased vessel wall stiffness, myocardial ischemia, heart failure, and increased cardiovascular morbidity and mortality. Unfortunately, there are currently no drugs available to treat MAC. The natural polyphenol epigallocatechin-3-gallate (EGCG) has been demonstrated to protect against cardiovascular disease; however, whether EGCG supplementation inhibits MAC in CKD remains unclear. In this study, we utilize a CKD-associated MAC model to investigate the effects of EGCG on vascular calcification and elucidate the underlying mechanisms involved. Our findings demonstrate that EGCG treatment significantly reduces calcium phosphate deposition and osteogenic differentiation of VSMCs *in vivo* and *in vitro* in a dose-dependent manner. In addition, through RNA sequencing (RNA-seq) analysis, we show a significant activation of the transcription factor JunB both in CKD mouse arteries and in osteoblast-like VSMCs. Notably, EGCG effectively suppresses CKD-associated MAC by inhibiting the activity of JunB. In addition, overexpression of JunB can abolish while knockdown of *JunB* can enhance the inhibitory effect of EGCG on the osteogenic differentiation of VSMCs. Furthermore, EGCG supplementation inhibits MAC in CKD via modulation of the JunB-dependent Ras/Raf/MEK/ERK signaling pathway. In conclusion, our study highlights the potential therapeutic value of EGCG for managing CKD-associated MAC, as it mitigates this pathological process through targeted inactivation of JunB.

## Introduction

In recent decades, the incidence of cardiovascular disease has steadily increased, rendering it the primary cause of death for numerous ethnic groups globally [1]. Vascular calcification plays a pivotal role in the pathogenesis of cardiovascular disease by causing ectopic deposition of calcium hydroxyapatite minerals in the arterial wall [ 1, 2] . Previous studies have shown that patients with vascular calcification associated with chronic kidney disease (CKD) have a significant increase in major adverse cardiovascular events [3]. Depending on where calcium phosphate deposits occur, vascular calcification is typically classified as intimal or medial calcification. Although both types are present in CKD patients, previous studies have demonstrated that mortality is more closely linked to medial arterial calcification (MAC) [4]. Therefore, identifying drugs to inhibit MAC or uncovering its molecular mechanisms is crucial for intervening in CKD.

Recent studies indicated that the osteogenic differentiation of vascular smooth muscle cells (VSMCs) is essential in this CKD-associated MAC [5]. When exposed to pathological stimuli, such as calcium and phosphate metabolism disorders, VSMCs undergo a phenotypic switch from a contracted state to an osteogenic phenotype [1]. These osteoblast-like VSMCs are capable of secreting various osteogenic differentiation proteins, including alkaline phosphatase (ALP), runt-related transcription factor 2 (Runx2), and bone morphogenetic protein-2 (BMP2), to promote the progression of MAC in CKD [6]. However, the precise pathophysiological mechanisms underlying the osteogenic differentiation of VSMCs remain poorly defined, and effective approaches for inhibiting the osteogenic differentiation of VSMCs are still lacking.

Epigallocatechin-3-gallate (EGCG) is a monomeric polyphenol compound extracted from green tea and is the primary component responsible for the pharmacological effects of green tea. EGCG has a variety of biological activities. EGCG has been recognized for its pharmacological effects on inflammatory bowel disease (IBD) due to its anti-inflammatory properties [7]. EGCG has antioxidant and free radical scavenging activities and is thus beneficial against metabolic diseases such as non-alcoholic fatty liver disease, obesity, and type 2 diabetes [8]. EGCG selectively induces the death of cancer cells by modulating antioxidant pathways, which can improve antitumor effects [9]. Numerous studies have shown that EGCG plays an active role in treating cardiovascular disease by attenuating acute myocardial infarction [10], improving cardiac hypertrophy [11] and heart failure [12], and exerting antiatherosclerotic effects [13]. Our previous study revealed that EGCG effectively reduces vitamin D3 (VitD3)-induced acute vascular calcification in mice [14]. However, its role in CKD-associated MAC has not been investigated.

In this study, RNA sequencing (RNA-seq) analysis and subsequent analyses of CKD mouse and VSMC calcification models revealed that the transcription factor JunB is a key factor in promoting MAC and that EGCG is most likely to inhibit MAC in CKD by inactivating JunB. JunB is one of the main activator protein-1 (AP-1) proteins in mammalian cells and has been identified as an essential contributor to the development of vascular diseases such as thoracic aortic aneurysm and aortic dissection [15]. JunB is also a key molecule that regulates the proliferation and migration of VSMCs [16]. However, whether JunB regulates the MAC in CKD remains unclear. We also used a CKD mouse model and a high-phosphate and calcium-induced VSMC model to investigate whether EGCG treatment prevents the progression of MAC in CKD patients and further investigated the underlying mechanisms involved. This study offers new insight into the therapeutic management of CKD-associated MAC.

## Materials and Methods

### Reagents

Epigallocatechin-3-gallate (EGCG, purity: ≥ 98%), CaCl _2_, 3-(4,5-dimethylthiazol-2-yl)-2,5-diphenyl tetrazolium bromide (MTT), 4′6-diamidino-2-phenylindole (DAPI), Alizarin Red S solution, a nuclear protein extraction kit, and a von Kossa kit were obtained from Solarbio Technology (Beijing, China). Beta-glycerophosphate (β-GP) was purchased from Sigma-Aldrich (St Louis, USA). The MEK inhibitor PD98059 and the Raf inhibitor sorafenib were obtained from APExBIO (Houston, USA). Primary human aortic smooth muscle cells (HASMCs; Cat. #6110) and smooth muscle cell medium were purchased from ScienCell Research Laboratories (Carlsbad, USA). All cell culture materials were obtained from Gibco (Grand Island, USA). RIPA lysis buffer, a BCA protein assay kit, an EdU staining kit, an ALP activity assay kit, and a Lipofectamine 8000 kit were obtained from Beyotime (Shanghai, China). A calcium assay kit was procured from Jiancheng Bioengineering Institute (Nanjing, China). The JunB overexpression plasmid, vector plasmid, small interfering RNAs (siRNAs) against human JunB (siJunB), and scrambled siRNA (siNC) were designed and synthesized by GenePharma (Shanghai, China). Trizol reagent was obtained from Life Technologies (Carlsbad, USA). Reverse transcription reagent and SYBR-Green PCR Master Mix were purchased from Accurate Biology (Changsha, China).

### Animals

Male C57BL/6J mice at 10 weeks of age and weighing 25 g to 30 g were purchased from Chengdu DOSSY Experimental Animal Co., Ltd. (Chengdu, China). The animal experiments were approved by the Animal Experimental Ethical Inspection of Sichuan University (No. K2021015). The mice were housed at the Laboratory Animal Center of Sichuan University (Chengdu, China) in a temperature-controlled room with a 12-h light/12-h dark cycle and free access to water and food.

In the CKD-associated MAC model, mice were randomly divided into 4 groups ( *n*  = 12/group): the sham group, CKD group, CKD + EGCG-low dose (25 mg/kg/d) group, and CKD + EGCG-high dose group (50 mg/kg/d). To induce CKD, the mice underwent standard two-stage 5/6 nephrectomy. Briefly, two-thirds of the mice’s left kidney was resected, and one week later, the entire right kidney was removed. The sham operation group underwent a sham operation in which the appropriate kidney was exposed and mobilized but not subjected to any other treatment. One week after the operation, the mice were fed with a 1.8% phosphorus diet for 8 weeks. For the EGCG intervention groups, the mice were given low or high doses of EGCG by gavage for 8 weeks starting from the first day of intake of a 1.8% phosphorus diet. For the sham and CKD groups, the mice were given 0.9% normal saline by gavage for 8 weeks starting from the first day of intake of a 1.8% phosphorus diet.

### Cell culture and treatments

HASMCs were cultured in smooth muscle cell medium. HASMCs from passages 4 to 11 were used in this study. HASMCs were incubated with calcifying medium (CM, growth medium supplemented with 10 mM β-GP and 3 mM CaCl _2_) and cultured at 37°C in an incubator containing 5% CO _2_ for 2‒21 days to induce cell calcification model. Different concentrations (0.1, 1, 5, 20, 30, 60, 90, or 100 μM) of EGCG were added to the HASMCs, and the medium was changed every 2 days. The control HASMCs were treated with growth medium (smooth muscle cell medium without 10 mM β-GP or 3 mM CaCl _2)_, and the growth medium was also changed every 2 days.

### Cell viability assay

HASMCs were seeded into 96-well plates at a density of 6×10 ^3^ cells/well and cultured in growth medium. The cells were pretreated with EGCG (0‒100 μM) for 2 days, after which MTT reagent was added to the growth medium at a concentration of 5 mg/mL. After 4 h of incubation, the supernatant was removed, and 100 μL of DMSO was added to each well. The 96-well plates were shaken to homogenize the liquid in the plates, and the optical density (OD) of each well was measured at 490 nm.

### Plasmid and siRNA transfection

According to the protocol provided by the manufacturer, specific plasmids and siRNAs (50 nM) were transfected into HASMCs using Lipofectamine 8000 reagent for 2 days. The sequences of the siRNAs used are listed in Supplementary Table S1.

### Quantitative real-time PCR

Total RNA was isolated from HASMCs using Trizol reagent. Then, 5× Evo M-MLV RT Reaction Mix was used for reverse transcription of the mRNA into cDNA. Quantitative real-time polymerase chain reaction (qRT-PCR) was performed with 2× Taq SYBR Green qPCR Mix, and detected using a CFX96 real-time PCR detection system (Bio-Rad, Hercules, USA). The data were collected, and the relative mRNA fold changes were calculated according to the 2 ^‒ΔΔCt^ method, with the housekeeping gene *GAPDH* serving as a reference. The primer sequences for the target genes are shown in Supplementary Table S2.

### Immunofluorescence staining

For immunofluorescence staining, HASMCs or mouse abdominal aorta sections were fixed with 4% paraformaldehyde for 20 min and permeabilized with 0.1% Triton X-100 (in PBS) for 15 min. After another 3 washes with PBS, the specimens were incubated with 5% goat serum for 1 h to block nonspecific binding. Afterward, the cells/tissues were incubated with primary antibodies overnight at 4°C. After being washed with PBS 3 times, the specimens were incubated with secondary antibodies for 1 h at room temperature, followed by incubation with DAPI for 10 min to stain the nuclei, and the specimens were subsequently washed in PBS 3 times. For F-actin staining, HASMCs were fixed with 4% paraformaldehyde for 15 min and then permeabilized with 0.1% Triton X-100 in PBS for 15 min at room temperature. Next, the cells were incubated with rhodamine phalloidin for 1 h at room temperature. Finally, images were obtained using a confocal laser-scanning microscope (LSM 710; Carl Zeiss, Wetzlar, Germany). The details of the antibodies used are listed in Supplementary Table S3.

### Western blot analysis

After 3 times wash with PBS, HASMCs and arterial tissues were lysed with cold RIPA lysis buffer supplemented with proteinase and phosphatase inhibitors. An enhanced BCA protein assay kit was used to measure the protein concentrations according to the manufacturer’s guidelines. Equal amounts of protein (20‒40 μg) were separated by sodium dodecyl sulfate-polyacrylamide gel electrophoresis (SDS-PAGE) and transferred to polyvinylidene fluoride (PVDF) membranes. The membranes were cut horizontally and blocked with 5% skim milk powder in TBST [tris-buffered saline (TBS) containing 0.1% Tween-20] for 2 h at room temperature. The membranes were incubated with primary antibody overnight at 4°C. The membranes were rinsed with TBST three times (10 min each) and then incubated with HRP-conjugated anti-rabbit secondary antibodies for 2 h at room temperature. The blots were visualized and detected with an imaging system. The protein band intensities were quantified by grayscale analysis with ImageJ software and normalized to the level of the GAPDH. A detailed list of antibodies with catalogue numbers and the corresponding companies is provided in Supplementary Table S3.

### EdU staining

HASMCs were incubated with 10 μM EdU staining buffer for 12 h. Subsequently, the cells were fixed in 4% polyformaldehyde for 15 min and permeabilized with 0.3% Triton X-100 (in PBS) for 10 min at room temperature. Then, the cells were coincubated with click additive solution for 30 min, and the nuclei were stained with Hoechst for 10 min at room temperature. Images of the cells were acquired using a confocal laser-scanning microscope (LSM 710). ImageJ software was used to determine the number of EdU-positive cells and the total number of nuclei. The percentage of EdU-positive cells was calculated as: (number of EdU-positive nuclei/total number of nuclei)×100%.

### Cell migration assay

HASMCs were seeded in 6-well culture plates and cultured with smooth muscle cell medium until they reached confluence. The HASMCs were then manually scratched with a 10-μL pipette tip to create a cell-free zone. The cells were gently washed with PBS to remove nonadherent cells and cultured in serum-free Dulbecco′s modified Eagle’s medium (DMEM) supplemented with 1% penicillin-streptomycin. Images of the scratched areas of the HASMCs were immediately taken using a Nikon microscope (Tokyo, Japan). Each group of cells was then incubated for 24 h after the indicated treatment, and a second set of images was taken after 24 h to measure HASMC migration over the scratched area. The cell-free area of each group was measured using ImageJ software and then analyzed according to the percentage of wound closure, which was calculated as the difference between the denuded area at 0‒24 h to the denuded area at 0 h.

### Alizarin Red S staining

For Alizarin Red S staining, HASMCs were seeded into 6-well plates. After 21 days of calcification induction, the HASMCs were fixed with 95% ethanol for 10 min and exposed to 1% Alizarin Red S solution (pH 4.2) for 10 min at room temperature. HASMCs were rinsed with ultrapure water three times to remove excess dye. The HASMCs that were positively stained displayed a reddish color, indicating calcification. For quantitative analysis, 10% formic acid (1 mL) was added to the 6-well plates to dissolve the Alizarin Red S dye, and the absorbance (405 nm) of the supernatant was measured using a microplate reader (SpectraMax 190, Molecular Devices, San Jose, USA).

For Alizarin Red S staining of whole mounts of the aorta, the aorta was fixed in 95% ethanol for 24 h and stained with 0.003% Alizarin Red S solution in 1% potassium hydroxide for 30 h at room temperature. The aortas were rinsed with 2% potassium hydroxide solution to remove excess dye from the surface of the tissue.

For Alizarin Red S staining of the mouse abdominal aorta sections, arterial segments were fixed in 4% formaldehyde, embedded in paraffin, and then cut into 5-μm-thick sections. The sections were deparaffinized, rehydrated, and then incubated with 1% Alizarin Red S staining solution for 30 min at room temperature. The sections were rinsed 3 times with ultrapure water to remove unbound dye. Images were captured with an Olympus Slideview VS200 (Olympus, Tokyo, Japan).

### Von Kossa staining

To examine aortic calcification, mouse abdominal aorta sections were deparaffinized, rehydrated, incubated with 5% silver nitrate buffer and exposed to ultraviolet light for 30 min at room temperature. The sections were rinsed with ultrapure water and incubated with 5% sodium thiosulfate for 2 min. The cell nuclei were then stained with hematoxylin. An Olympus Slideview VS200 slide scanner (Olympus) was used to image the slides.

### ALP activity assay

After 7 days of HASMC culture, the proteins were extracted by sonicating the HASMCs with 1% Triton X-100 in 0.9% saline on ice. The supernatants were collected by centrifugation at 10,000 *g* for 5 min. ALP activity in the supernatants was determined using an ALP activity assay kit. After the protein concentration was tested with a BCA protein assay kit, the ALP activity was normalized to the total protein concentration.

### Calcium content quantification

To quantify the precipitated calcium, after the HASMCs were cultured for 7 days, the medium was removed from the 6-well plates, and the HASMCs were washed with PBS. Then, 500 μL of 0.6 M HCl was added to each well, and the HASMCs were decalcified for 24 h at 4°C. The supernatants were collected, and the calcium content in the supernatants was assessed using a calcium assay kit. For calcium content analysis of mouse aortas, the aorta was cut into small segments of ~2 mm and then incubated with 0.6 M HCl for 48 h at 4°C. The calcium content in the supernatants was quantified with a calcium assay kit. After determining the protein concentration in the supernatants using the BCA protein assay kit, the calcium content in the HASMCs or mouse aortas was normalized to the total protein concentration.

### Nuclear/cytoplasmic protein extraction

After 7 days of culture, the HASMCs were washed 3 times with PBS. According to the manufacturer’s recommendations, nuclear and cytoplasmic protein lysates were extracted from HASMCs using a nuclear protein extraction kit.

### Statistical analysis

Data are presented as the mean±standard error of the mean (SEM). Statistical analysis was performed with GraphPad Prism (version 8.4.3). After checking for normality and equal variance, the differences between more than two groups were compared using one-way ANOVA followed by Tukey’s post hoc test. *P*  < 0.05 was considered to indicate statistical significance.

## Results

### EGCG attenuates MAC in CKD mice

**Figure 1:**
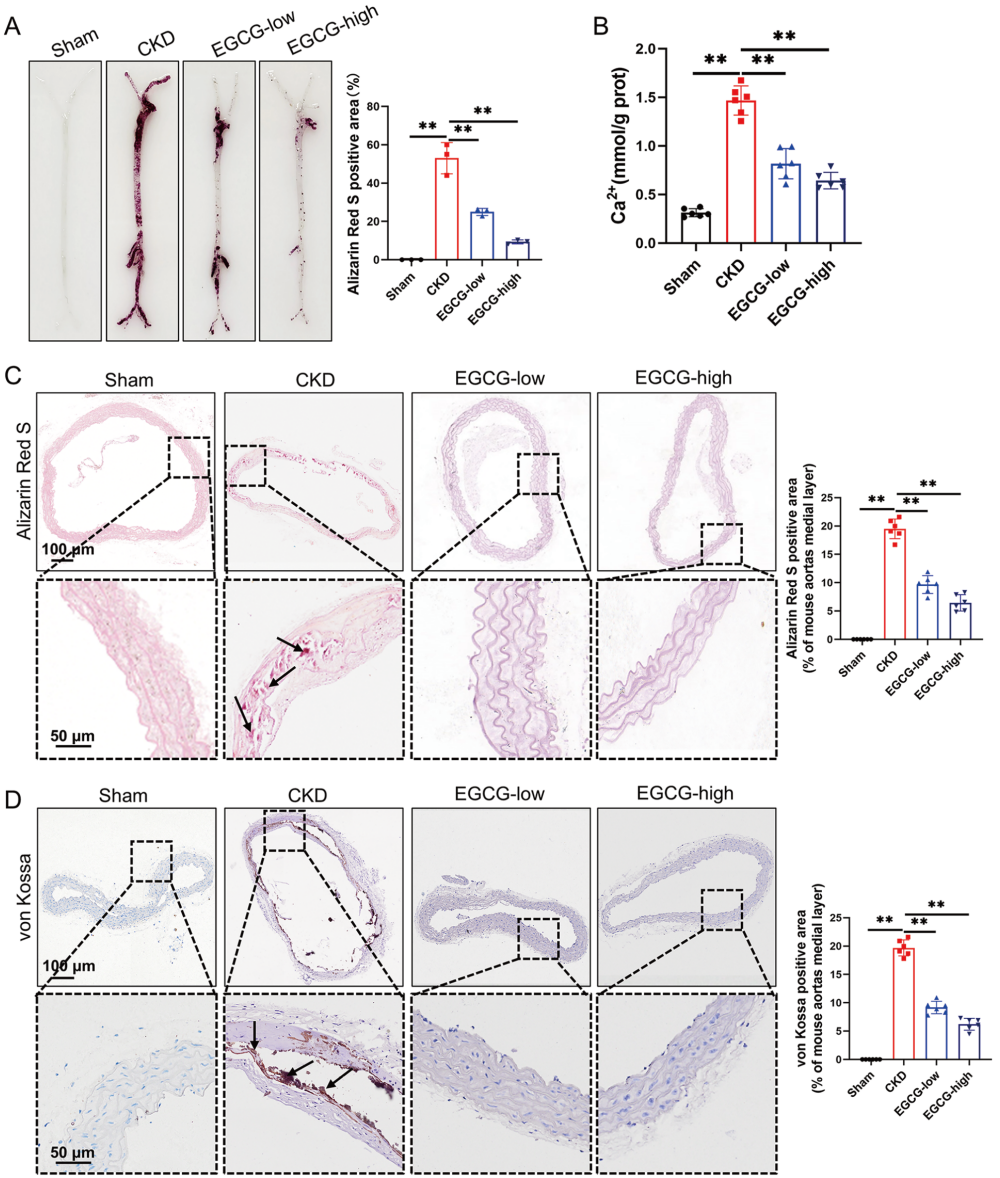
Figure 1 EGCG attenuates mineral deposition in CKD mouse aortas (A) Mineral deposition in the CKD mouse aortas was assessed by Alizarin Red S staining. (B) Calcium content in the mouse aortas was measured. (C,D) Mineral deposition in mouse abdominal aorta sections was assessed by Alizarin Red S and von Kossa staining. Boxed areas at higher magnifications are shown below. Data are shown as the mean ± SEM. **P < 0.01 (n = 3 or 6).

Previous studies have shown that EGCG (40 mg/kg/d, i.g.) administration for 18 weeks markedly attenuated atherosclerotic plaque formation [13]. Both high- and low-dose EGCG (50 mg/kg/d and 20 mg/kg/d) markedly mitigated colonic inflammation in a murine inflammatory bowel disease model [17]. Therefore, we chose 50 mg/kg/d and 25 mg/kg/d doses of EGCG for the *in vivo* study. As shown in Figure 1A, Alizarin Red S staining of the aortas revealed decreased aortic mineral accumulation in the EGCG-treated groups compared with the model group. In addition, the high-dose EGCG had better pharmacological effects, as shown by the calcium content of the aortas ( Figure 1B). Moreover, Alizarin Red S and von Kossa staining of aorta sections further confirmed that both low and high doses of EGCG significantly reduced aortic mineral deposition in CKD mice ( Figure 1C,D).

**Figure 2:**
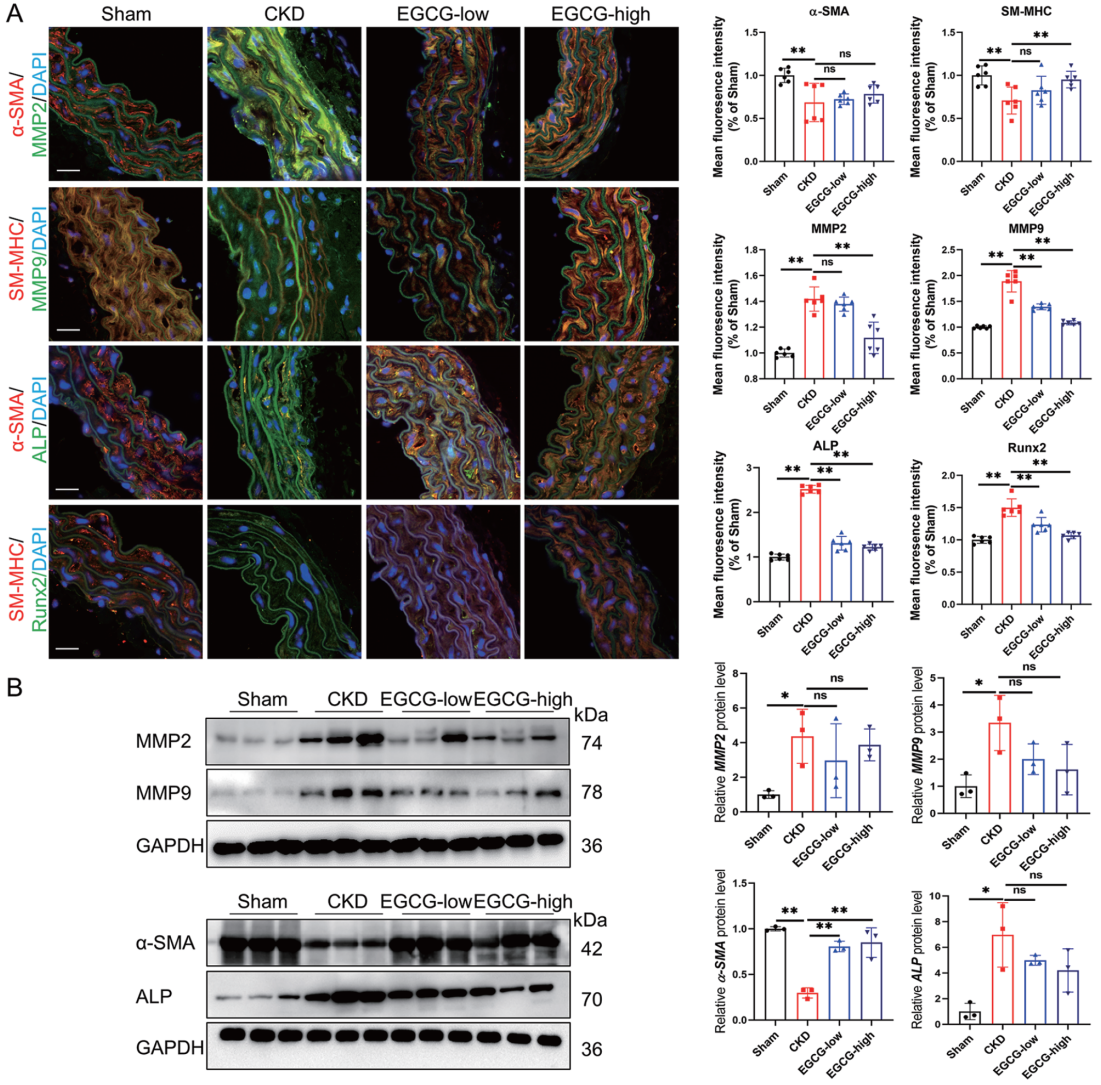
Figure 2 EGCG inhibits VSMC osteogenic differentiation in CKD mouse aortas (A) Immunofluorescence staining was used to determine the expressions of α-SMA, SM-MHC, MMP2, MMP9, ALP, and Runx2 in CKD mouse aortas. Scale bar: 20 μm. (B) MMP2, MMP9, α-SMA, and ALP expressions in mouse aortas were analyzed by western blot analysis. Data are shown as the mean ± SEM. *P < 0.05, **P < 0.01, ns: not significantly different between the indicated groups (n = 3 or 6).

The phenotypic switch of VSMCs from the contractile to the osteogenic phenotype is a critical process in the development of MACs [18]. Matrix metalloproteinase-2 (MMP2) and MMP9 can initiate MAC by participating in the migration of VSMCs, promoting the transdifferentiation of VSMCs into osteogenic cells, degrading elastic fibers, and regulating matrix remodeling [ 16, 19, 20] . Immunofluorescence staining revealed that the expressions of MMP2 and MMP9 and the expressions of VSMC osteogenic markers (ALP and Runx2) in CKD mouse aortas were significantly decreased after EGCG intervention ( Figure 2A). However, the VSMC contractile markers α-smooth muscle actin (α-SMA) and smooth muscle myosin heavy chain (SM-MHC) were significantly upregulated ( Figure 2A). In addition, western blot analysis further confirmed that low and high doses of EGCG could reverse the changes in the expressions of proteins associated with the osteogenic differentiation of VSMCs in CKD mouse aortas ( Figure 2B). These results suggested that EGCG can inhibit MAC by reducing aortic mineral deposition and regulating the osteogenic differentiation of VSMCs in CKD mouse aortas.

### EGCG attenuates calcification of VSMCs *in vitro*

**Figure 3:**
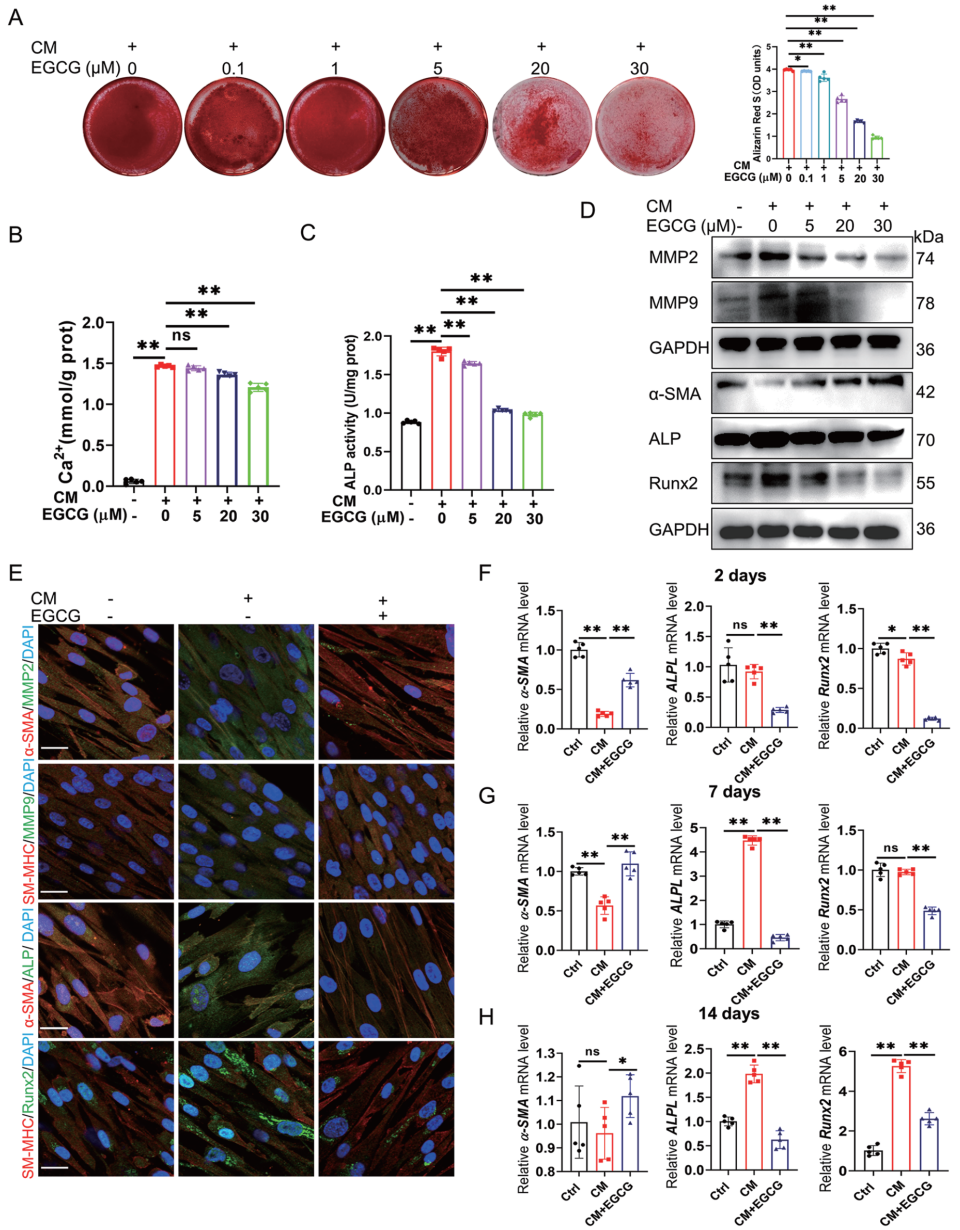
Figure 3 EGCG inhibits mineral deposition and the osteogenic differentiation of HASMCs in vitro (A) Mineral deposition in HASMCs was assessed by Alizarin Red S staining. (B,C) Calcium content and ALP activity were measured in HASMCs. (D) Western blot analysis of MAC-related proteins in HASMCs. (E) Immunofluorescence staining was used to determine the protein expressions of α-SMA, SM-MHC, MMP2, MMP9, ALP, and Runx2. Scale bar: 20 μm. (F‒H) After treatment with EGCG (20 μM) for 2, 7, and 14 days, the mRNA expressions of α-SMA, ALPL, and Runx2 in HASMCs were determined by qRT-PCR. Data are shown as the mean ± SEM. *P < 0.05, **P < 0.01, ns: not significantly different between the indicated groups (n = 5).

Furthermore, we investigated the effect of EGCG on the calcification of VSMCs *in vitro*. First, different concentrations (0, 0.1, 1, 5, 20, 30, 60, 90, and 100 μM) of EGCG were co-incubated with HASMCs for 2 days. MTT assays confirmed that 0‒100 μM EGCG did not change the cell viability of HASMCs ( Supplementary Figure S1). After various concentrations of EGCG were incubated with HASMCs in CM for 21 days, Alizarin Red S staining indicated that mineral deposition was significantly reduced when HASMCs were treated with 5, 20, and 30 μM EGCG ( Figure 3A). Subsequently, calcium content and ALP activity assays confirmed that EGCG concentration-dependently reduced calcium deposition and ALP activity in HASMCs ( Figure 3B,C). Furthermore, we examined the influence of EGCG on the osteogenic differentiation of HASMCs *in vitro*. Western blot analysis demonstrated that EGCG treatment inhibited the osteogenic differentiation of VSMCs, as evidenced by the upregulation of α-SMA and the downregulation of MMP2, MMP9, ALP, and Runx2 in HASMCs ( Figure 3D and Supplementary Figure S2). All the above research results confirmed that 20 μM EGCG could effectively inhibit the osteogenic differentiation of HASMCs. Therefore, a dose of 20 μM was chosen for subsequent experiments. Immunofluorescence images showed that EGCG treatment downregulated the expressions of MMP2, MMP9, and ALP, inhibited the nuclear translocation of Runx2, and increased the expressionss of α-SMA and SM-MHC ( Figure 3E). Moreover, the qRT-PCR results also showed that EGCG enhanced the mRNA expression of *α-SMA*, reducing the expressions of *ALP* and *Runx2* in HASMCs at different calcification stages ( Figure 3F‒H). These data demonstrated that EGCG inhibits the mineralization and osteogenic differentiation of VSMCs under pro-calcification conditions.

### EGCG inhibits the activation of JunB *in vivo* and *in vitro*

**Figure 4:**
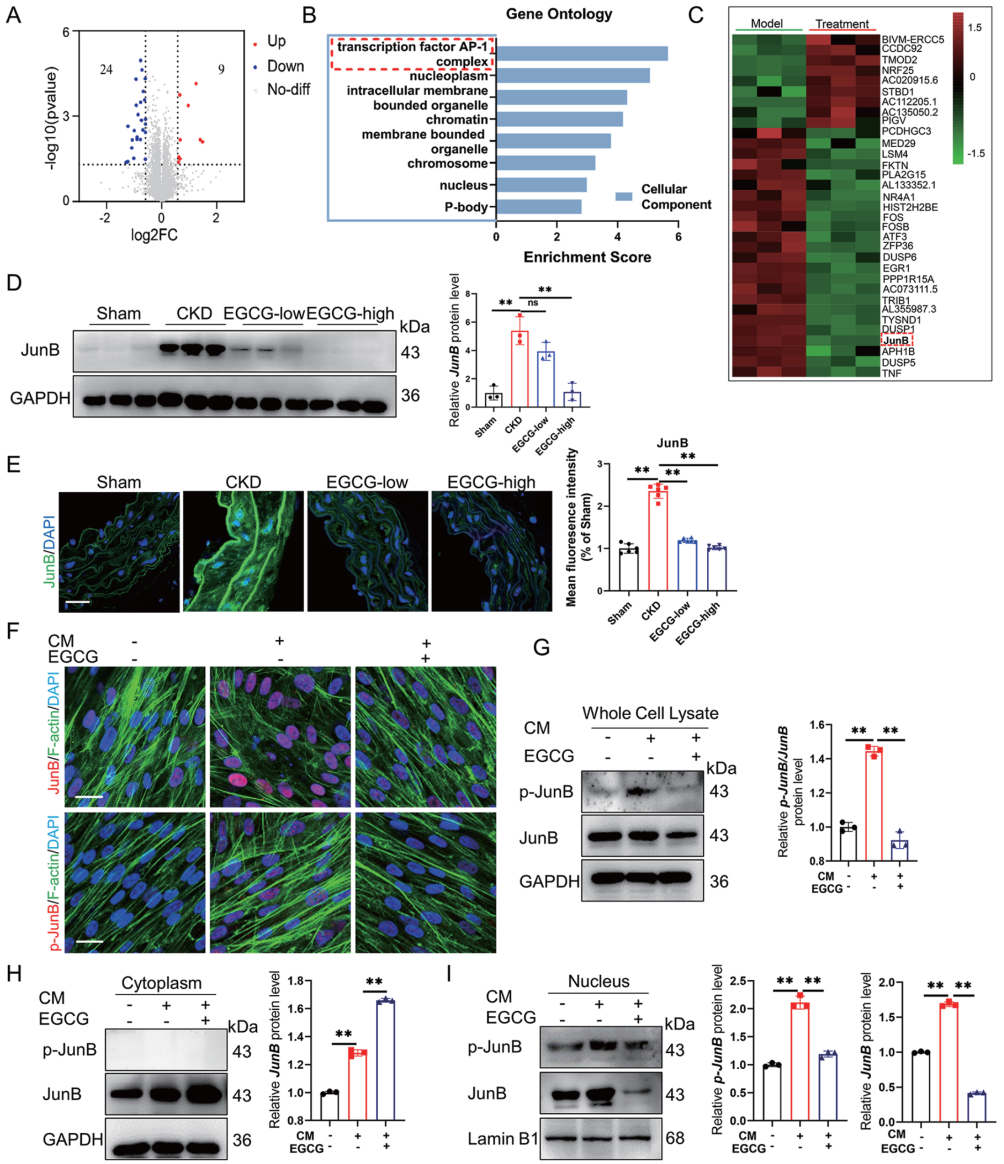
Figure 4 EGCG inhibits the activation of JunB in vivo and in vitro (A) The volcano plot shows the number of differentially expressed genes (DEGs) downregulated or upregulated after EGCG treatment. Genes with a fold change greater than 1.5 and a Q value < 0.05 were considered DEGs. FC indicates the fold change. (B) GO analysis of the model and EGCG treatment groups. (C) DEG Heatmap of the model and EGCG treatment groups. (D) JunB protein expression levels in CKD mouse aortas were analyzed by western blot analysis. (E) Immunofluorescence staining of JunB distribution and expression in vivo after EGCG treatment. Scale bar: 20 μm. (F) Immunofluorescence staining was used to detect the distribution and expressions of JunB and p-JunB in HASMCs. Scale bar: 20 μm. (G‒I) Western blot analysis of JunB and p-JunB in HASMCs in whole-cell lysates, the cytoplasm, and the nucleus. Data are shown as the mean ± SEM. **P < 0.01, ns: not significantly different between the indicated groups (n = 3 or 6).

To explore the mechanism underlying the inhibitory effects of EGCG on MAC, we conducted RNA-seq analysis of HASMCs induced with CM (model) for 2 days. As shown in Figure 4A, the expressions of 11,920 genes were significantly changed after EGCG treatment. Among these genes, 33 exhibited more than 1.5-fold changes, with 24 downregulated and 9 upregulated genes ( Figure 4A). Gene Ontology (GO) functional enrichment analysis revealed that genes affected by EGCG treatment were highly enriched in GO terms related to the transcription factor AP-1 complex ( Figure 4B). In addition, the gene Heatmap results showed that the expression of JunB, an important transcription factor of the AP-1 family, was significantly upregulated ( Figure 4C). Therefore, we focused on JunB and verified the effects of EGCG on the expression and transcription of JunB *in vivo* and *in vitro*. First, we detected the protein expression level of JunB in CKD mouse aortas by western blot analysis. As shown in Figure 4D, both low and high doses of EGCG significantly reduced the expression of JunB in CKD-associated MAC mouse aortas. Moreover, the immunofluorescence staining results confirmed that EGCG administration significantly reduced JunB expression and nuclear transcript levels *in vivo* ( Figure 4E). Furthermore, immunofluorescence staining also demonstrated that EGCG treatment markedly decreased JunB nuclear translocation and phosphorylated JunB (p-JunB) expression in CM-induced HASMCs ( Figure 4F). Moreover, western blot analysis results further confirmed that EGCG treatment could alter JunB and p-JunB levels in the cytoplasm and nucleus, as evidenced by the downregulation of JunB and p-JunB in the nucleus ( Figure 4G‒I).

### EGCG suppresses osteogenic differentiation of VSMCs by inactivating JunB

**Figure 5:**
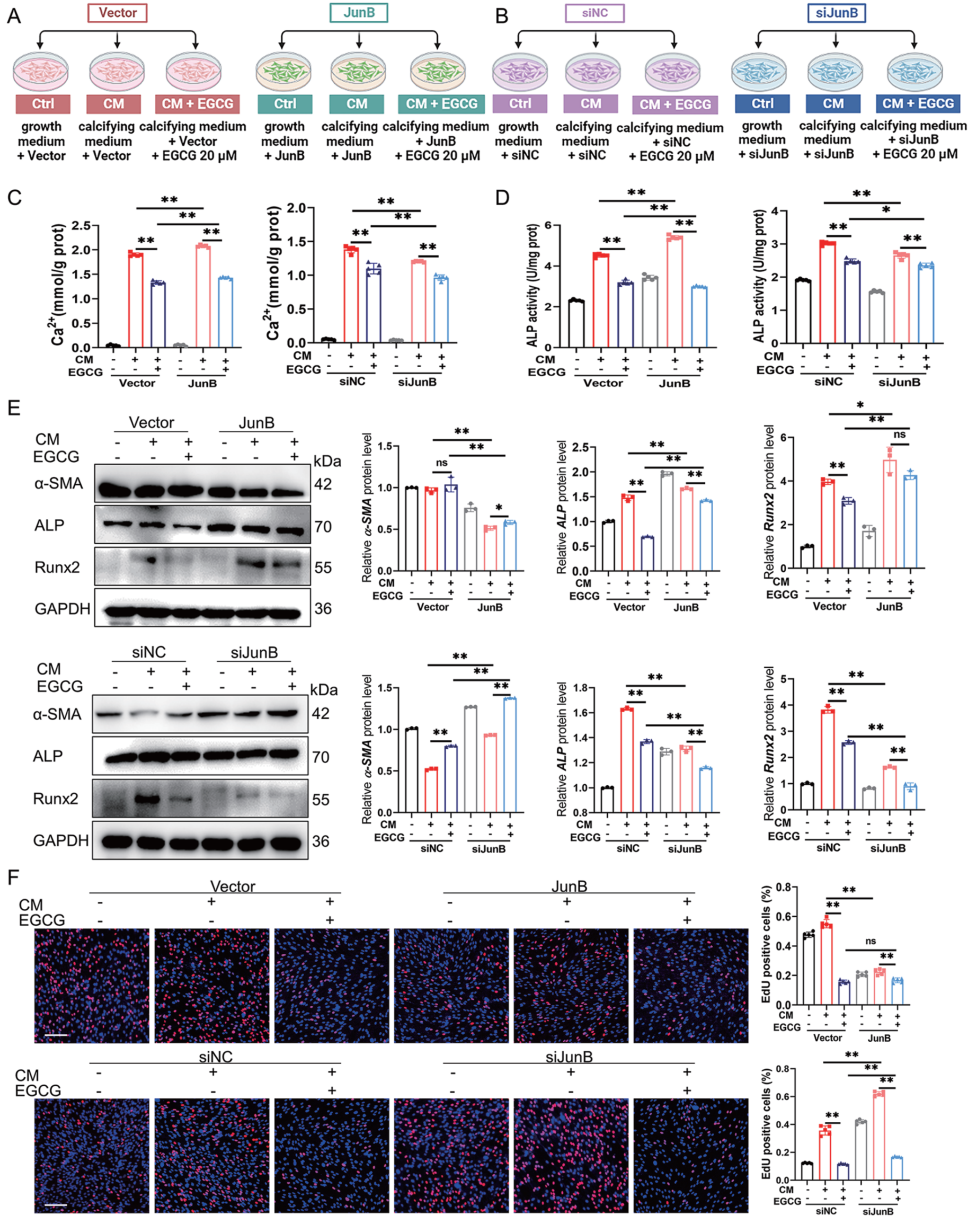
Figure 5 EGCG improves MAC by downregulating JunB (A,B) Schematic diagrams of the experimental design for HASMCs with JunB overexpression or knockdown that was co-treated with EGCG. (C,D) Calcium content and ALP activity were measured in HASMCs. (E) Western blot analysis of MAC-related proteins in HASMCs. (F) The results of the EdU staining assay and quantification. Representative EdU-positive cells (red) and Hoechst (blue) are shown. Scale bar: 200 μm. Data are shown as the mean ± SEM. **P < 0.01, ns: not significantly different between the indicated groups (n = 3 or 5).

To determine the role of JunB in the EGCG-mediated inhibition of MAC, we investigated whether the overexpression or knockdown of *JunB* could affect the mineralization and osteogenic differentiation of HASMCs. The expression level of JunB was detected by western blot analysis to verify the success of the overexpression plasmid construction ( Supplementary Figure S3A). Simultaneously, HASMCs were transfected with three siRNAs targeting JunB or a negative control siRNA. Western blot analysis results indicated that the second siRNA strongly reduced the expression of JunB ( Supplementary Figure S3B). Schematic diagrams of the experimental design for HASMCs with JunB overexpression or knockdown are shown in Figure 5A,B. Calcium content and ALP activity assays revealed that JunB overexpression markedly blocked the inhibitory effect of EGCG on HASMC calcification, resulting in further increases in mineral deposition and ALP activity in HASMCs under pro-calcification conditions ( Figure 5C,D). Moreover, overexpression of JunB upregulated the protein expression of ALP and Runx2 and decreased the expression of α-SMA ( Figure 5E and Supplementary Figure S4). However, the knockdown of *JunB* enhanced the inhibitory effects of CM-induced osteogenic differentiation in HASMCs caused by EGCG treatment ( Figure 5E and Supplementary Figure S4). In addition, we examined the effect of JunB on the proliferation of HASMCs in the presence of EGCG. EdU staining showed that EGCG significantly reduced the proliferation of HASMCs with JunB overexpression or knockdown ( Figure 5F). These results indicated that JunB is a key molecule involved in the EGCG-mediated inhibition of MAC and that the overexpression of JunB blocked the effect of EGCG on the osteogenic differentiation of HASMCs.

### EGCG attenuates MAC by inactivating the Ras/Raf/MEK/ERK signaling pathway

**Figure 6:**
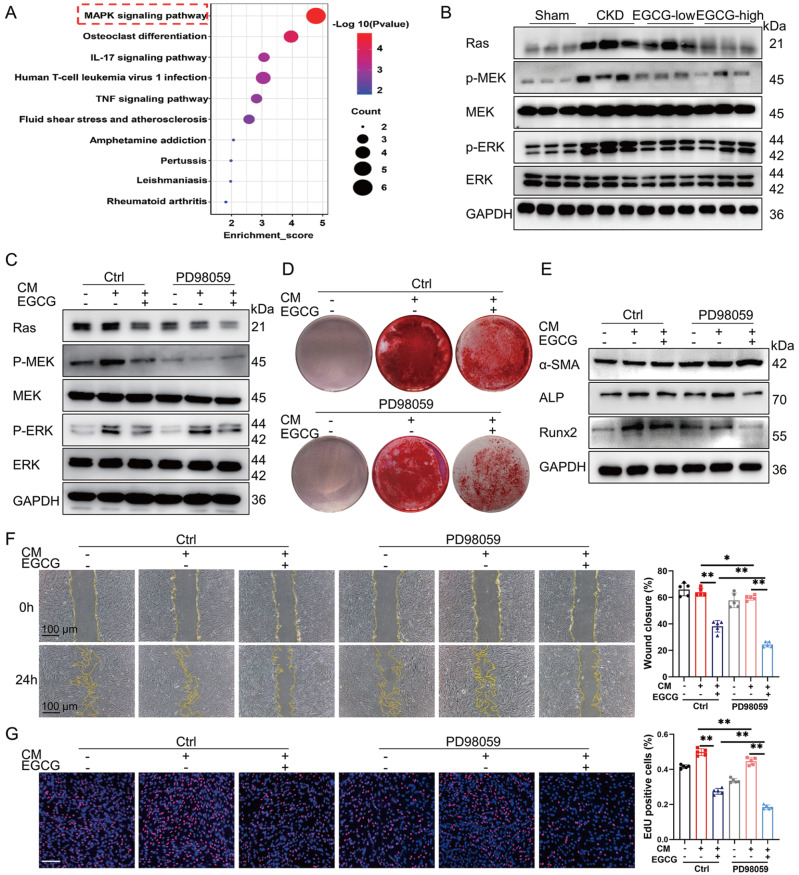
Figure 6 EGCG attenuates MAC by inactivating the Ras/Raf/MEK/ERK signaling pathway (A) The results of KEGG pathway analysis. (B) Western blot analysis of the Ras/Raf/MEK/ERK signaling pathway markers in CKD mouse aortas. (C) Western blot analysis of Ras/Raf/MEK/ERK signaling pathway markers in HASMCs after co-incubation with EGCG and PD98059. (D) Alizarin Red S staining was performed to examine mineral deposition in HASMCs. (E) Western blot analysis of MAC-related proteins in HASMCs. (F) Representative phase-contrast images of HASMC migration in the scratch assay and quantification. (G) Images of EdU-stained cells and their quantification. Scale bar: 200 μm. Data are shown as the mean ± SEM. *P < 0.05, **P < 0.01, ns: not significantly different between the indicated groups (n = 5).

KEGG pathway analysis revealed that EGCG modulated the MAPK signaling pathway ( Figure 6A). The mitogen-activated protein kinase (MAPK) pathway, often known as the Ras/Raf/MEK/ERK signal cascade, is vital for regulating physiological processes [21]. We evaluated the expressions of the key proteins Ras, MEK, and ERK, which are markers of the Ras/Raf/MEK/ERK signaling pathway. Western blot analysis results revealed that Ras, p-MEK, and p-ERK expressions significantly increased in CKD mouse aortas ( Figure 6B and Supplementary Figure S5A). However, this effect was blocked by EGCG treatment ( Figure 6B and Supplementary Figure S5A). In addition, to better understand the role of EGCG in this pathway, we used PD98059 (10 μM), an inhibitor of this signaling pathway. An *in vitro* study confirmed that EGCG and PD98059 attenuated CM-mediated Ras/Raf/MEK/ERK signaling pathway marker expression in HASMCs ( Figure 6C and Supplementary Figure S5B). Subsequently, we further explored the role of the Ras/Raf/MEK/ERK signaling pathway in the EGCG-mediated inhibition of MAC. Alizarin Red S staining demonstrated that the EGCG-mediated alleviation of mineral deposition was increased by the presence of PD98059 ( Figure 6D and Supplementary Figure S5C). Western blot analysis results also confirmed that co-treatment with PD98059 significantly increased the inhibitory effect of EGCG on the osteogenic differentiation of VSMCs ( Figure 6E and Supplementary Figure S5D). In addition, we found that co-treatment with EGCG and PD98059 significantly reduced the migration and proliferation of HASMCs ( Figure 6F,G). These results indicated that EGCG ameliorates MAC by inactivating the Ras/Raf/MEK/ERK signaling pathway both *in vivo* and *in vitro*.

### EGCG reduces the transcription of JunB via the Ras/Raf/MEK/ERK signaling pathway

**Figure 7:**
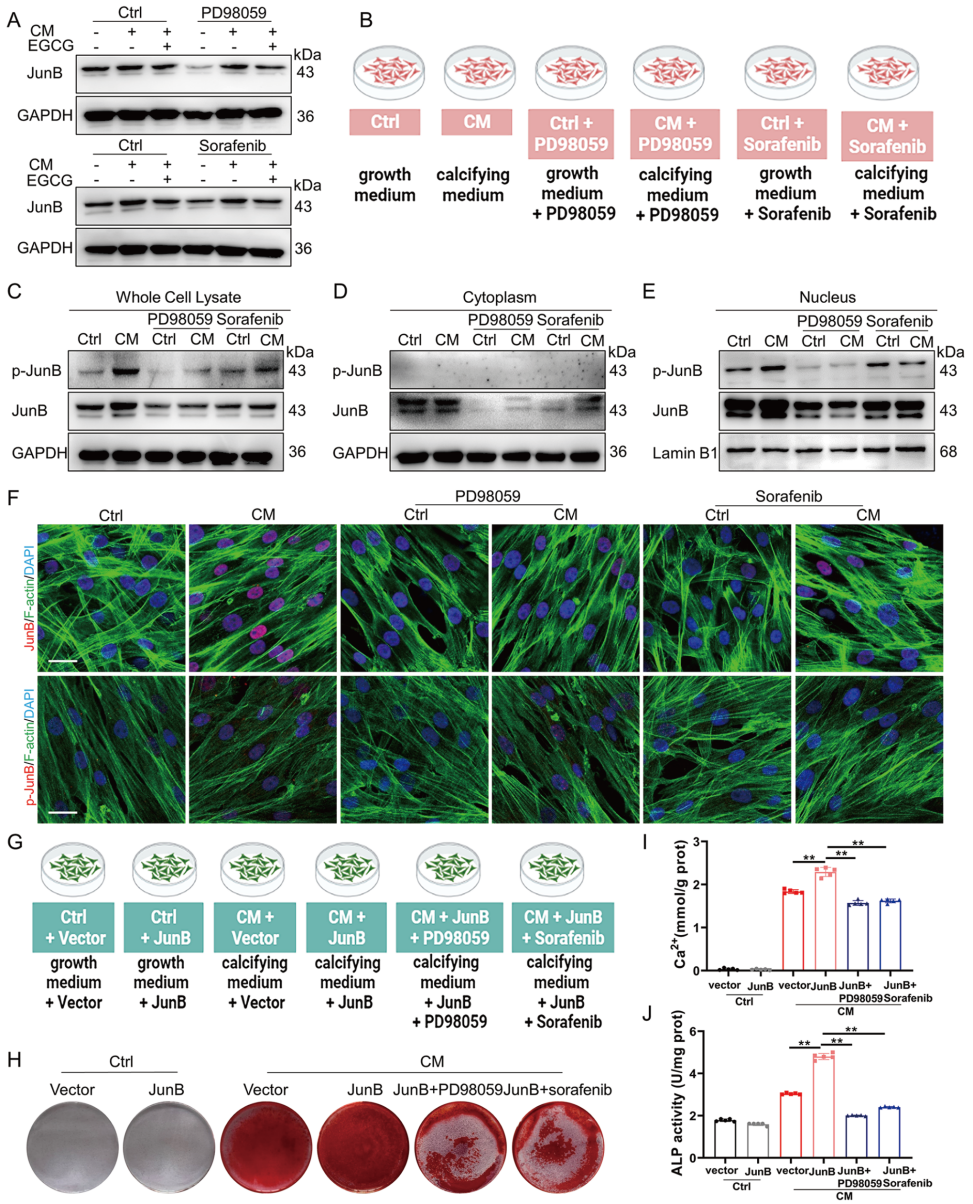
Figure 7 The Ras/Raf/MEK/ERK signaling pathway is involved in the EGCG-mediated inactivation of JunB (A) Western blot analysis of JunB after treatment with EGCG and Ras/Raf/MEK/ERK signaling pathway inhibitors (PD98059 and sorafenib). (B) A schematic diagram of the experimental design of HASMCs cotreated with inhibitors. (C‒E) Western blot analysis of JunB and p-JunB in whole-cell lysates, cytoplasm, and nuclei. (F) Immunofluorescence staining was used to examine the distribution and expressions of JunB and p-JunB in HASMCs. Scale bar: 20 μm. (G) A schematic diagram of the experimental design of JunB-overexpressing HASMCs co-treated with inhibitors. (H) Calcium deposition in HASMCs was detected by Alizarin Red S staining. (I,J) Calcium content and ALP activity were measured in HASMCs. Data are shown as the mean ± SEM. **P < 0.01 (n = 5).

To further explore the relationship between JunB and the Ras/Raf/MEK/ERK signaling pathway in the EGCG-mediated inhibition of MAC, we co-incubated HASMCs with Ras/Raf/MEK/ERK signaling pathway inhibitors (PD98059 and sorafenib (200 nM)) and EGCG. Under pro-calcification conditions, the inhibitors and EGCG significantly decreased JunB expression in HASMCs ( Figure 7A and Supplementary Figure S6A). A schematic diagram of the experimental design of HASMCs co-treated with inhibitors is shown in Figure 7B. Supplementation with inhibitors reduced the protein expressions of JunB and p-JunB ( Figure 7C and Supplementary Figure S6B). Interestingly, under pro-calcification conditions, the inhibitors reduced JunB expression in the cytoplasm, decreased JunB nuclear translocation, and decreased p-JunB expression in the nucleus ( Figure 7D,E and Supplementary Figure S6C,D). Immunofluorescence staining results also showed that the Ras/Raf/MEK/ERK signaling pathway inhibitors reduced JunB nuclear translocation and decreased p-JunB expression ( Figure 7F). A schematic diagram of the experimental design for JunB-overexpressing HASMCs co-treated with inhibitors is shown in Figure 7G. Alizarin Red S staining, calcium content, and ALP activity assays showed that JunB-mediated HASMC calcification was attenuated by PD98059 and sorafenib ( Figure 7H‒J and Supplementary Figure S6E). These results suggested that inactivation of the Ras/Raf/MEK/ERK signaling pathway could inhibit JunB nuclear translocation and block JunB-mediated calcification. These results indicated that the Ras/Raf/MEK/ERK signaling pathway participates in the inactivation of JunB mediated by EGCG.

## Discussion

**Figure 8:**
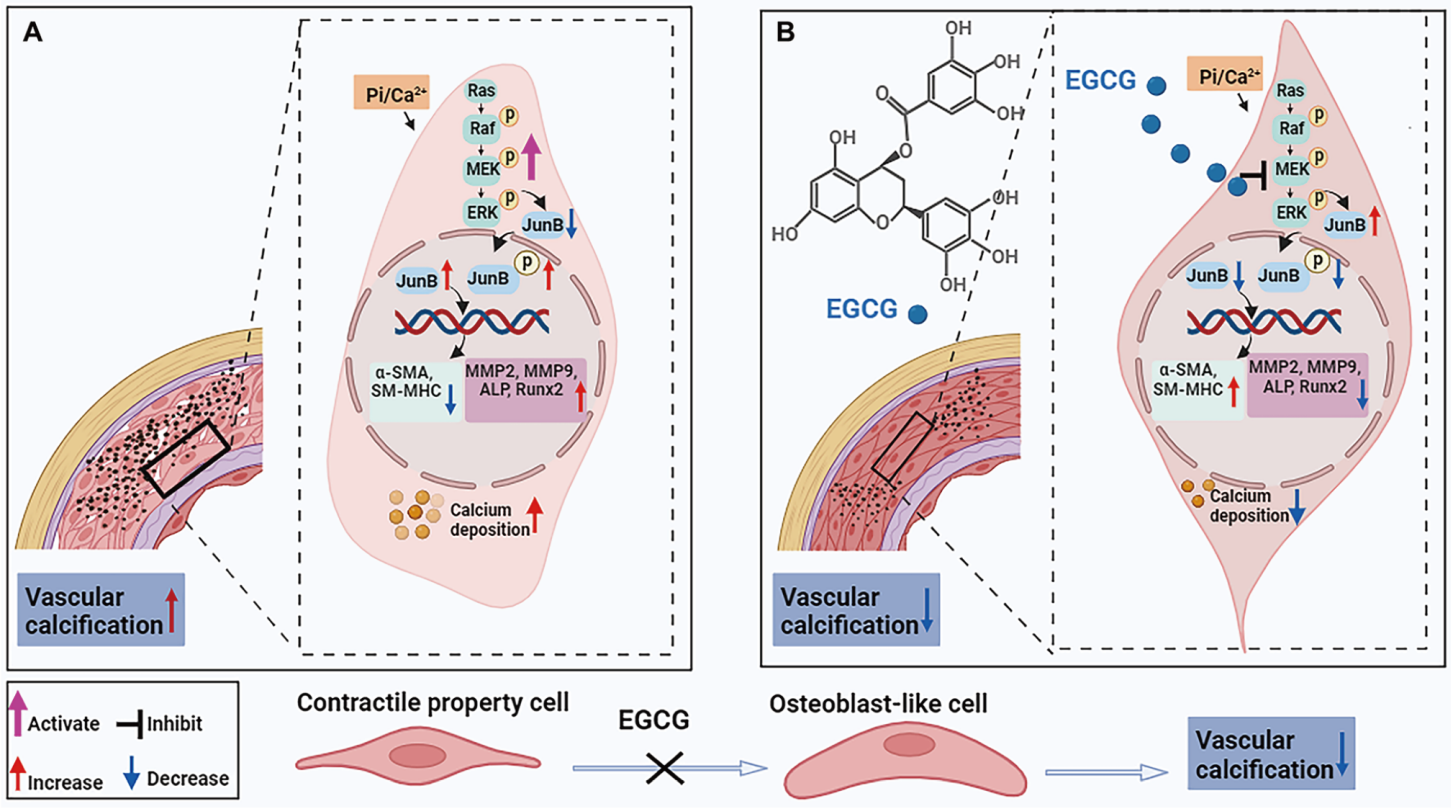
Figure 8 A schematic diagram of the mechanism by which EGCG inhibits MAC (A) The Ras/Raf/MEK/ERK signaling pathway in VSMCs is activated under high phosphorus and calcium conditions, which promotes the nuclear translocation of JunB and increases the expression of p-JunB. These changes leads to a decrease in contractile phenotype markers (α-SMA and SM-MHC) and an increase in osteogenic differentiation-associated MMP2, MMP9, ALP, and Runx2. (B) After EGCG treatment, the Ras/Raf/MEK/ERK signaling pathway is inactivated; JunB expression is increased in the cytoplasm, while JunB nuclear translocation and p-JunB expression are decreased; contractile phenotype markers are increased, and osteogenic differentiation-related proteins are decreased.

MAC is an important pathological basis for cardiovascular disease and a major risk determinant for cardiovascular mortality, which is common in CKD patients [ 22, 23] . However, the intricate molecular mechanisms involved in CKD-associated MAC have limited the development of efficacious therapeutic approaches [24]. This study investigated whether EGCG, a natural polyphenol, alleviates MAC in CKD patients. Interestingly, we demonstrated that EGCG dose-dependently attenuated calcium deposition and inhibited the osteogenic differentiation of VSMCs both *in vivo* and *in vitro*. Furthermore, our findings indicated that JunB is strongly downregulated in response to EGCG treatment, highlighting the pivotal role of JunB in accelerating MAC in CKD. Mechanistically, the anti-MAC effect of EGCG is manifested by inactivation of JunB, which is dependent on the Ras/Raf/MEK/ERK signaling pathway ( Figure 8).

Many studies have shown that EGCG can protect the cardiovascular system by decreasing vascular inflammation, oxidative stress, and cell apoptosis. Together with warfarin, EGCG significantly ameliorates thrombosis and prevents endothelial cell damage via the PI3K/AKT and extracellular signal-regulated kinase (ERK) pathways [25]. EGCG protects against oxidized low-density lipoprotein (ox-LDL)-induced endothelial cell damage and attenuates atherosclerosis via the Jagged-1/Notch signaling pathway [26]. However, the specific molecular mechanism by which EGCG inhibits MAC in CKD is unclear.

JunB is a basic leucine zipper (bZIP) protein that forms the AP-1 complex by dimerization with other bZIP proteins, such as Fos or basic leucine zipper ATF-like transcription factor (BATF) family members [27]. Recent studies have revealed that JunB plays a critical role in the development of cardiovascular diseases. Anxa1 can maintain the contractile phenotype of VSMCs by binding to JunB and activating myosin light chain 9 (MYL9) [28]. The cyclic peptide cyclo-(PheTyr), which is isolated from Sparganii Rhizoma, can alleviate ischaemic stroke reperfusion brain injury via the JunB/JNK/nuclear factor-kappaB (NF-κB) signaling pathway [29]. MicroRNA-663 can regulate VSMC phenotypic switching and vascular neointimal formation by targeting the JunB/MYL9 signaling pathway [16]. To date, the association between JunB and MAC in CKD remains unknown. Our findings demonstrated that JunB is significantly activated in CKD mouse aortas and osteoblast-like VSMCs. As anticipated, JunB overexpression increased the expression of osteogenic markers in HASMCs. Furthermore, *JunB* knockdown reduced the progression of MAC. In addition, our study revealed considerable increases in both the nuclear translocation and phosphorylation of JunB in HASMCs under pro-calcification conditions. These results demonstrate that JunB is involved in the progression of MAC. Additionally, overexpression of JunB decreased the inhibitory effect of EGCG on the osteogenic differentiation of VSMCs, while the knockdown of *JunB* further enhanced the inhibitory effect of EGCG. In conclusion, our findings indicate that the inactivation of JunB is critical for the inhibitory effect of EGCG on MAC and that EGCG may alleviate the osteogenic differentiation of VSMCs in a JunB-dependent manner.

MAC in CKD is a multifactorial and intricately regulated phenomenon governed by complex intracellular signaling pathways [30]. In this study, we conducted RNA-seq analysis and discovered that after EGCG supplementation, the Ras/Raf/MEK/ERK signaling pathway was markedly decreased. The Ras/Raf/MEK/ERK signaling pathway plays an important role in regulating the phenotypic switch of VSMCs [31]. Previous studies have shown that EGCG can potentially manage cancer by modulating the Ras/Raf/MEK/ERK signaling pathway [ 32, 33] . In this study, the protein expression levels of Ras/Raf/MEK/ERK signaling pathway markers in CKD mouse aortas and osteoblast-like VSMCs were increased. Nonetheless, EGCG supplementation significantly reduced the expression of Ras and the phosphorylation of MEK and ERK. Therefore, to probe the relationship between the Ras/Raf/MEK/ERK signaling pathway and MAC, we used PD98059, a classic inhibitor of MEK. We found that inhibition of this pathway significantly reversed the changes in osteogenic differentiation-related protein levels and decreased mineral deposition and the proliferation of HASMCs, which were further reduced by EGCG supplementation. These results prove that the Ras/Raf/MEK/ERK signaling pathway is involved in EGCG-mediated MAC.

Moreover, we further investigated the relationship between JunB and the Ras/Raf/MEK/ERK signaling pathway. We found that the distribution of JunB in HASMCs could be altered by pharmacological inhibition of Raf1 and MEK. Under pro-calcification conditions, PD98059 and sorafenib decreased JunB nuclear translocation and reduced p-JunB expression in the nucleus. Furthermore, these inhibitors reduced mineral deposition, calcium content, and ALP activity in JunB-overexpressing HASMCs, providing compelling evidence that the Ras/Raf/MEK/ERK signaling pathway is involved in JunB-mediated MAC.

Nevertheless, there are several limitations in this study. First, the effects of EGCG on age-matched female mice were not investigated. Therefore, the conclusions can only be generalized to males. Second, further experiments are needed to determine the effect of EGCG on arterial calcification by using *JunB* ^-^knockout mice to better understand the molecular mechanisms by which EGCG inhibits MAC. Finally, EGCG has several disadvantages, such as low stability in aqueous solution and low oral bioavailability *in vivo*, which limit its application in the treatment of several diseases [34]. Therefore, researchers have combined EGCG with liposomes or nanoparticles to prepare a variety of novel EGCG-based complex materials, such as EGCG nanoliposomes and Fe-EGCG nanocomplexes, which have greatly improved the bioavailability of EGCG [ 35, 36] . In the future, we also expect to find suitable EGCG nanocomplexes to improve the efficacy of EGCG in the treatment of MAC.

New therapeutic targets and drugs are urgently needed to treat and prevent MAC in CKD patients. We demonstrated that JunB is a key regulator of MAC and that EGCG protects against MAC by inactivating JunB. EGCG level can be increased by dietary intervention or pharmacological approaches. EGCG (200 to 300 mg) is present in brewed cups (240 mL) of green tea [37]. Epidemiological studies have indicated that green tea consumption of more than 3‒4 cups/day could reduce the risk of cardiovascular disease and all-cause mortality [ 38, 39] . This study indicated that drinking several cups of green tea daily or taking a similar dose of EGCG tablets reduced the risk of MAC.

In conclusion, our study suggested that EGCG protects against CKD-associated MAC *in vivo* and *in vitro*. Additionally, we identified the activation of JunB in VSMCs as an essential cause of MAC in CKD. Finally, we demonstrated that EGCG can inhibit JunB activation by regulating the Ras/Raf/MEK/ERK signaling pathway. Our results elucidate the mechanism by which EGCG inhibits CKD-related MAC and provide a reference for the clinical translational application of EGCG.

## Supporting information

24166Supplementary_Data

## Supplementary Data

Supplementary data is available at *Acta Biochimica et Biophysica Sinica* online.
