# Supplementary material for: Epigallocatechin-3-gallate inhibits osteogenic differentiation of vascular smooth muscle cells through the transcription factor JunB: Epigallocatechin-3-gallate inactivates the transcription factor JunB
Source: Acta Biochim Biophys Sin (Shanghai). 2024 Jun 3;57(6):901–15. doi: 10.3724/abbs.2024060 (PMC12247136; doi:10.3724/abbs.2024060)
Supplement: 24166Supplementary_Data [file 24166Supplementary_Data.pdf]

Supplementary Figures

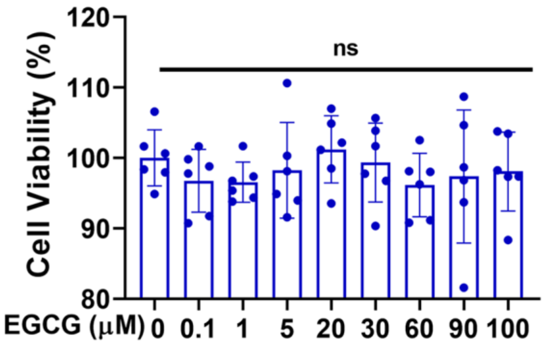

Supplementary Figure S1. Effect of EGCG on the viability of HASMCs HASMCs were treated with serially diluted EGCG for 2 days, and cell viability was measured by MTT assay. Data are expressed as the mean ± SEM. ns: not significantly different between indicated groups (*n*=6).

For Figure 3D

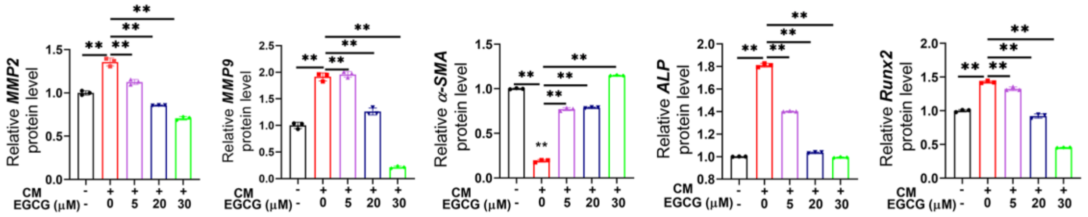

Supplementary Figure S2. Statistical analysis of western blots in Figure 3D Data are expressed as the mean ± SEM. *P<0.05, **P<0.01, ns: not significantly different between indicated groups (n=3).

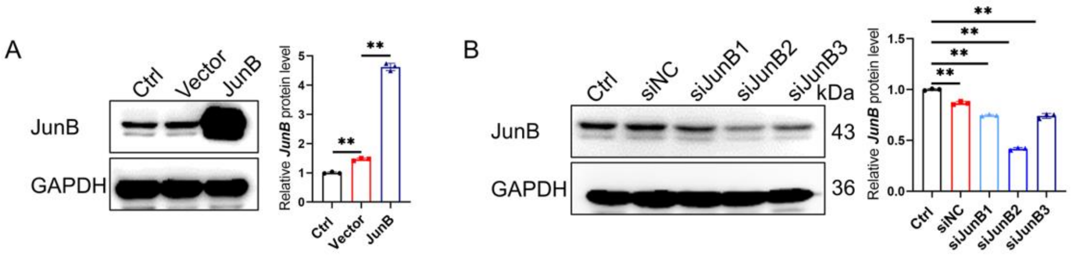

Supplementary Figure S3. Validation of successful construction of *JunB* overexpression or knockdown HASMCs (A) HASMCs were transfected with vector plasmid and JunB overexpression plasmid for 2 days. Western blot analysis of JunB-overexpression in HASMCs. (B) HASMCs were transfected with three different siRNAs targeting JunB or negative control. Western blot analysis of JunB in HASMCs. Data are shown as the mean ± SEM. *P<0.05, **P < 0.01 (n=3).

For Figure 5E

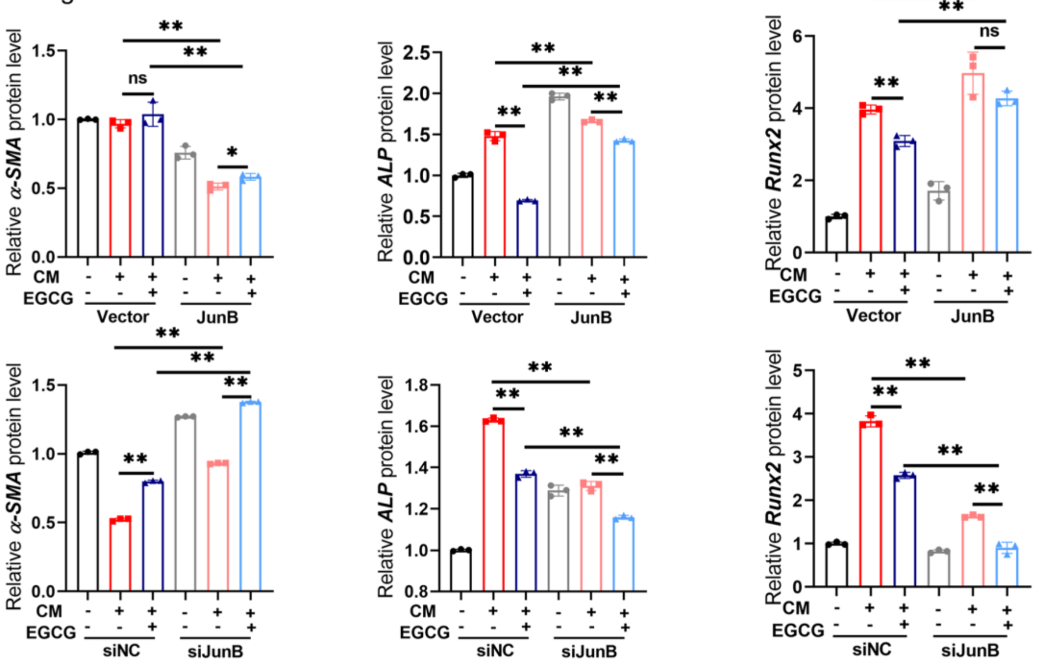

Supplementary Figure S4. Statistical analysis of western blots in Figure 5E Data are expressed as the mean ± SEM. *P<0.05, **P<0.01. ns: not significantly different between indicated groups (n =3).

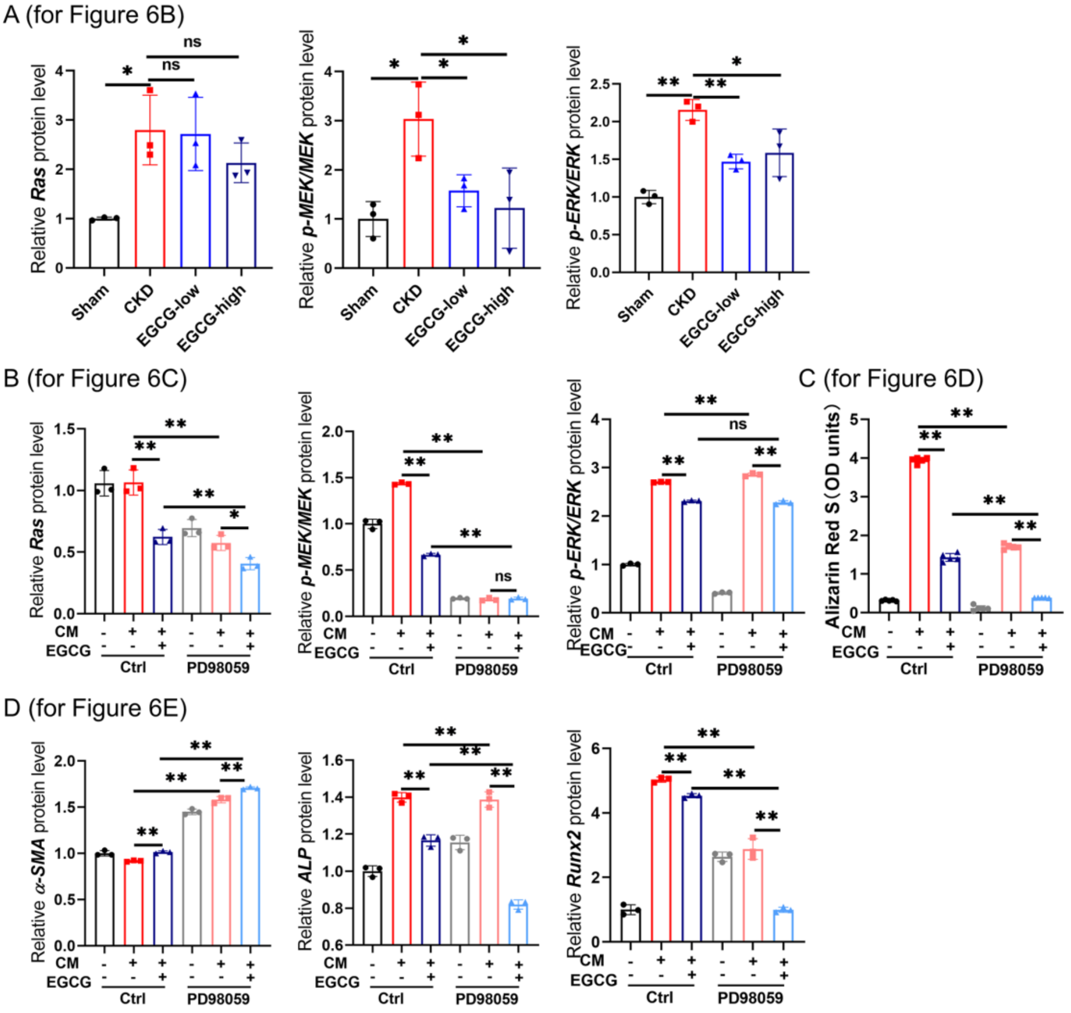

Supplementary Figure S5. Statistical analysis of Alizarin Red S staining and western blots in Figure 6 (A) Figure 6B. (B) Figure 6C. (C) Figure 6D. (D) Figure 6E. Data are expressed as the mean ± SEM. **P*<0.05, ***P*<0.01. ns: not significantly different between indicated groups (n =3 or 5).

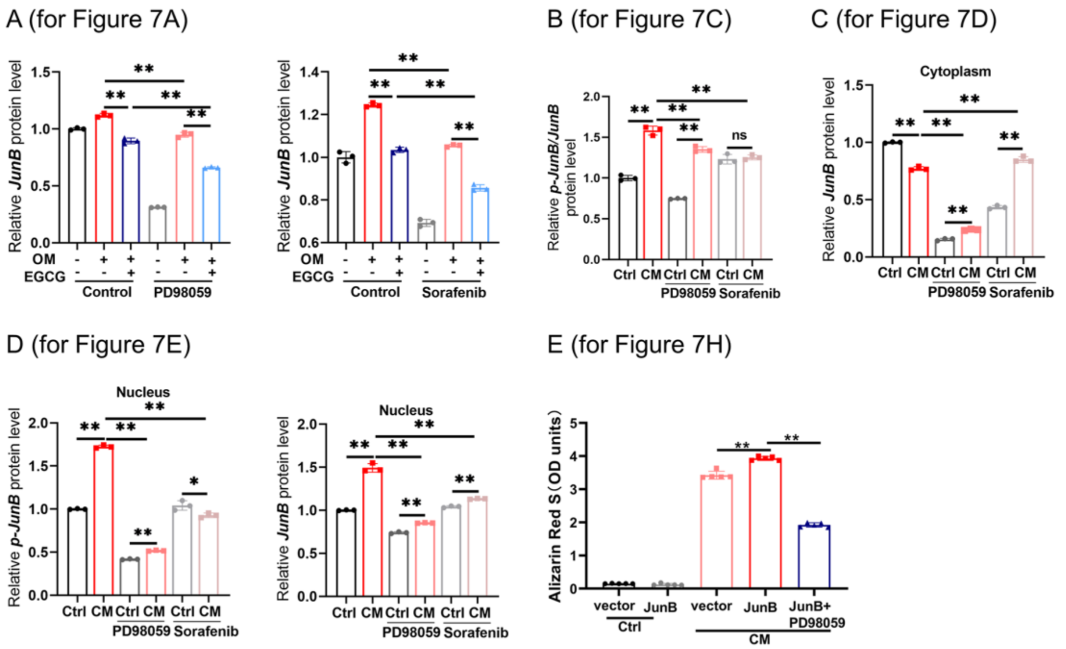

Supplementary Figure S6. Statistical analysis of Alizarin Red S staining and western blots in Figure 7 (A) Figure 7A. (B) Figure 7C. (C) Figure 7D. (D) Figure 7E. (E) Figure 7H. Data are expressed as the mean ± SEM. *P<0.05, **P< 0.01 (n=3 or 5).

Supplementary Table S1. siRNAs used in this work

Name	Sequences (5′→3′)
Negative control sense	UUCUCCGAACGUGUCACG
Negative control antisense	ACGUGACACGUUCGGAGAATT
siJunB-1 sense	GACGACUCAUACACAGCUATT
siJunB-1 antisense	UAGCUGUGUAUGAGUCGUCTT
siJunB-2 sense	CACAAGAUGAACCACGUGATT
siJunB-2 antisense	UCACGUGGUUCAUCUUGUGTT
siJunB-3 sense	GUUUACACCAACCUCAGCATT
siJunB-3 antisense	UGCUGAGGUUGGUGUAAACTT

Supplementary Table S2. The sequences of primers for human genes used in this work

Gene	Primer sequence (5′→3′)
α-SMA	Forward: GTGTTGCCCCTGAAGAGCAT
	Reverse: GCTGGGACATTGAAAGTCTCA
ALPL	Forward: ACTGGTACTCAGACAACGAGAT
	Reverse: ACGTCAATGTCCCTGATGTTATG
Runx2	Forward: TGGTTACTGTCATGGCGGGTA
	Reverse: TCTCAGATCGTTGAACCTTGCTA
GAPDH	Forward: ACAACTTTGGTATCGTGGAAGG
	Reverse: GCCATCACGCCACAGTTTC

Supplementary Table S3. The antibodies used in this study

Designation	Source and identifiers	Dilution ratio
Anti-JunB	SAB #49169	WB 1:1000; IF 1:100
Anti-Phospho-JunB	SAB #11027	WB 1:1000; IF 1:100
Anti-α-SMA (Rabbit)	CST #19245	WB 1:1000
Anti-α-SMA (Mouse)	SAB #40482	IF 1:100
Anti-SM-MHC	Proteintech #60222-1-Ig	IF 1:100
Anti-MMP2	Abcam #ab92536	WB 1:1000; IF 1:100
Anti-MMP9	Abcam #ab283575	WB 1:1000; IF 1:100
Anti- ALP	HUABIO #ET1601-21	WB 1:1000; IF 1:100
Anti- Runx2	CST #D1L7F	WB 1:1000; IF 1:100
Anti-Ras	HUABIO #ET1601-16	WB 1:1000
Anti-phospho-MEK1/2	CST #9154	WB 1:1000
Anti-MEK1/2	CST #9126	WB 1:1000
Anti-phospho-ERK1/2	CST#4370	WB 1:1000
Anti-ERK1/2	CST #4695	WB 1:1000
Anti-GAPDH	SAB #41549	WB 1:3000
Anti-Lamin B1	CST #13435	WB 1:1000
Goat anti-rabbit IgG secondary antibody	SAB #L3012	WB 1:10 000
